# Insulin Icodec Weekly: A Basal Insulin Analogue for Type 2 Diabetes

**DOI:** 10.17925/EE.2023.19.1.4

**Published:** 2023-03-16

**Authors:** Harpreet S Bajaj, Ronald M Goldenberg

**Affiliations:** 1. LMC Diabetes & Endocrinology, Brampton, Ontario, Canada; 2. LMC Diabetes & Endocrinology, Concord, Ontario, Canada

**Keywords:** Insulin icodec, basal, weekly, type 2 diabetes

## Abstract

Insulin icodec is a once-weekly basal insulin analogue in late-phase clinical development. Similar efficacy and safety of icodec to once-daily basal insulin analogues have been reported in over 4,200 participants with type 2 diabetes from three phase II and five phase III trials. Indeed, glycated haemoglobin reduction was superior for icodec among insulin-naïve participants (ONWARDS 1, 3 and 5) and in those switching from a daily basal insulin in ONWARDS 2, with the latter trial demonstrating improved diabetes treatment satisfaction scores with insulin icodec versus insulin degludec.

Insulin remains an important diabetes treatment, with 150–200 million people worldwide requiring insulin therapy.^1^ While insulin is vital for managing type 1 diabetes, basal insulin is typically recommended for type 2 diabetes when non-i nsulin therapies are not enough to achieve glycaemic targets.^2^ Several barriers related to basal insulin therapy for type 2 diabetes contribute to non-achievement of glycaemic targets, including delay of insulin initiation or titration, needle phobia with daily injections, missed insulin doses, insulin discontinuation and hypoglycaemia.^3^

The availability of once-weekly insulin formulations may help overcome many of the barriers related to basal insulin, including the convenience of fewer injections, similarly to the advantage of once-weekly versus daily glucagon-like peptide-1 (GLP-1) receptor agonists.^4^ Given that about one-third of daily insulin-treated patients do not adhere to therapy, the availability of a once-weekly basal insulin formulation should aid in improving adherence.^3^^,^^5^ Currently, two once-weekly insulin formulations are in late-phase clinical development: basal insulin Fc (insulin efsitora alfa) and insulin icodec, which has now completed phase III trials.

Icodec is an analogue of human insulin, with three substitutions to the amino acid structure and an attached C20 icosane fatty diacid chain that allows the molecule to bind reversibly to albumin (similarly to insulin detemir), prolonging the half life to 196 hours (approximately 7 days) and achieving steady state after 3–4 once-weekly injections.^6^ One unit of icodec provides the same glucose lowering as one unit of comparator daily basal insulins, with an equivalent once-weekly dose being seven times that of a daily basal insulin.^7^ Three phase II randomized trials of icodec (700 U/mL) were conducted in patients with type 2 diabetes with comparator once-daily glargine 100 U/mL (U100). The pivotal double-blind, double-dummy trial enrolled insulin-naïve patients with type 2 diabetes who were inadequately controlled with metformin with or without a dipeptidyl peptidase-4 inhibitor. Participants who received icodec had a statistically comparable glycated haemoglobin (HbA1C) reduction, along with a similar incidence of combined level 2 (clinically significant hypoglycaemia defined as <3 mmol/L [<54 mg/dL]) and level 3 (severe) hypoglycaemia.^8^ In the second insulin-n aïve trial, a less intensive titration algorithm (i.e. slower weekly increments titrated to a less stringent target of 4.4–7.2 mmol/L [80–130 mg/dL]) reduced the risk for hypoglycaemia while maintaining adequate glycaemic control.^9^ In the third phase II trial, switching from once-daily to weekly insulin demonstrated the benefit of adding a one-time loading dose to the first calculated icodec dose (to help reach steady state faster than the typical 3–4 weeks) with improved continuous glucose monitoring (CGM) metrics of time in range and time above range during weeks 15 and 16 without a clinically significant increase in hypoglycaemia. Unlike the icodec group that did not receive a loading dose, the icodec participants treated with a loading dose did not have a rise in fasting glucose in the first 3 weeks following insulin initiation.^10^ Weight gain across the phase II trials ranged from 0.6 kg to 1.5 kg with insulin icodec, and was clinically similar to that of insulin glargine U100.^8-10^

Topline data from six phase IIIa, treat-to-target trials in the ONWARDS programme for icodec are summarized in *Figure 1*. HbA1C as the primary outcome was superior for icodec versus comparators in all three trials with insulin-naïve patients (ONWARDS 1, 3 and 5), as well as in ONWARDS 2 where patients were switched from a daily basal insulin.^11-14^ Insulin icodec was non-inferior to insulin glargine U100 in patients on basal-bolus insulin (ONWARDS 4).^13^ Notably, similar rates of combined level 2 and level 3 hypoglycaemia were observed in ONWARDS 1–5 in patients with type 2 diabetes; however, in patients with type 1 diabetes, this rate was higher for icodec compared with daily degludec at 26 weeks of the ONWARDS 6 trial, despite similar glycaemic control.^11-14^ While ONWARDS 6 results for the full duration of 52 weeks are awaited, it will be interesting to evaluate detailed CGM metrics, especially related to the timing of hypoglycaemia, in addition to a careful reassessment of the icodec dose titration algorithm for individuals with type 1 diabetes. Diabetes treatment satisfaction score was significantly improved with insulin icodec in ONWARDS 2, and better than for insulin degludec.^11^ Other eagerly awaited results from the ONWARDS programme include participant satisfaction and/or adherence scores in ONWARDS 5 and 6.

**Figure 1:**
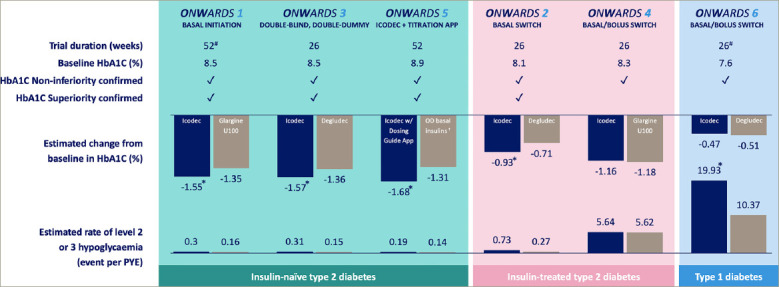
Insulin icodec phase IIIa programme (ONWARDS) topline results

*Statistically significant versus the comparator; ^†^Insulin degludec or insulin glargine U100/U300; # Primary results are presented. Note that the treatment arms in ONWARDS 1 were of 78 weeks' duration, while those in ONWARDS 6 were 52 weeks' duration.

*Clinically significant hypoglycaemia (level 2): blood glucose <3.0 mmol/L (<54 mg/dL). Severe hypoglycaemia (level 3): hypoglycaemia with severe cognitive impairment requiring external assistance for recovery.*

*HbA1C = glycated haemoglobin; OD = once daily; PYE = patient years of exposure; U100 = 100 U/mL.*

Once insulin icodec is approved for use, there will be some practical issues and concerns related to its clinical implementation. Based on the ONWARDS programme, patients with type 2 diabetes who are candidates for insulin icodec will include insulin-naïve patients and basal insulin-treated patients not achieving glycaemic targets, especially when adherence to daily injections is a concern. Further studies may be required before insulin icodec can be recommended in type 1 diabetes. For insulin-naïve individuals with type 2 diabetes, the ONWARDS programme suggests a starting dose of 70 U weekly (*Figure 2A*), which is equivalent to 10 U of a daily basal insulin with the same injection volume as 100 U/mL daily basal insulin due to 7× the concentration (700 U/mL). Titration is by 20 U weekly if fasting glucose is above or below target in the 3 days prior to the next injection (*Figure 2B*).^7^ When switching to insulin icodec from previously established basal insulin, the initial dose is 7× the previous daily dose of basal, with a one-time additional 50% of the calculated once-weekly dose (10.5× daily basal dose) (*Figure 2A*). At week 2, the recommended dose is 7× the previous daily dose, with on-going weekly titration by 20 U adjustments from week 3 (*Figure 2B*).^7^ Clinicians may decide to titrate less aggressively, perhaps 10 U weekly, for patients at greater risk of hypoglycaemia.

While the phase IIIa programme in type 2 diabetes demonstrated similar rates of level 2 or 3 hypoglycaemia compared with the currently available daily basal insulin analogues, there are clinical questions related to hypoglycaemia that need answering given the long duration of action of insulin icodec. Although CGM data from phase II demonstrated the duration of hypoglycaemic episodes was similar with icodec and glargine U100,^15^ it remains a clinical concern that the dose of icodec cannot be down-titrated more than once weekly, and it needs to be determined whether there is greater risk of recurrent hypoglycaemia following an episode while awaiting subsequent downtitration. A meticulous review of CGM hypoglycaemia duration metrics from the larger ONWARDS 2 and 4 trials, which enrolled participants with higher hypoglycaemia risk and longer duration of diabetes, might be helpful to allay these concerns. The impact of exercise frequency and intensity on hypoglycaemia in icodec-treated participants is an important area to explore in future research. Furthermore, clinical situations where hypoglycaemia may be more likely, especially when oral intake is reduced due to acute illness or a medical procedure/ surgery, may pose challenges for icodec dosing, both in terms of holding icodec doses or switching back to daily basal insulin temporarily, perhaps during hospitalization. CGM analyses from the post-treatment phases of the ONWARDS 1, 2 and 4 trials may provide guidance to help with these clinical scenarios.

As insulin icodec will likely be combined with other non-insulin anti-hyperglycaemic agents, a background-medication-specific analysis of the efficacy and safety data from the ONWARDS programme will help elucidate the effects of icodec when combined with different agents. For people with type 2 diabetes, there is also the exciting possibility of combining a weekly GLP-1 receptor agonist with a weekly insulin in the same injection. Icosema (Novo Nordisk, Bagsværd, Denmark), a fixed ratio combination of icodec and semaglutide, has recently started a phase IIIa programme.^16^ This combination has the potential to reduce injection burden from two to one injection per week, while further improving acceptability, gastrointestinal tolerability and adherence, with potential for weight loss and reduced risk of hypoglycaemia.

**Figure 2:**
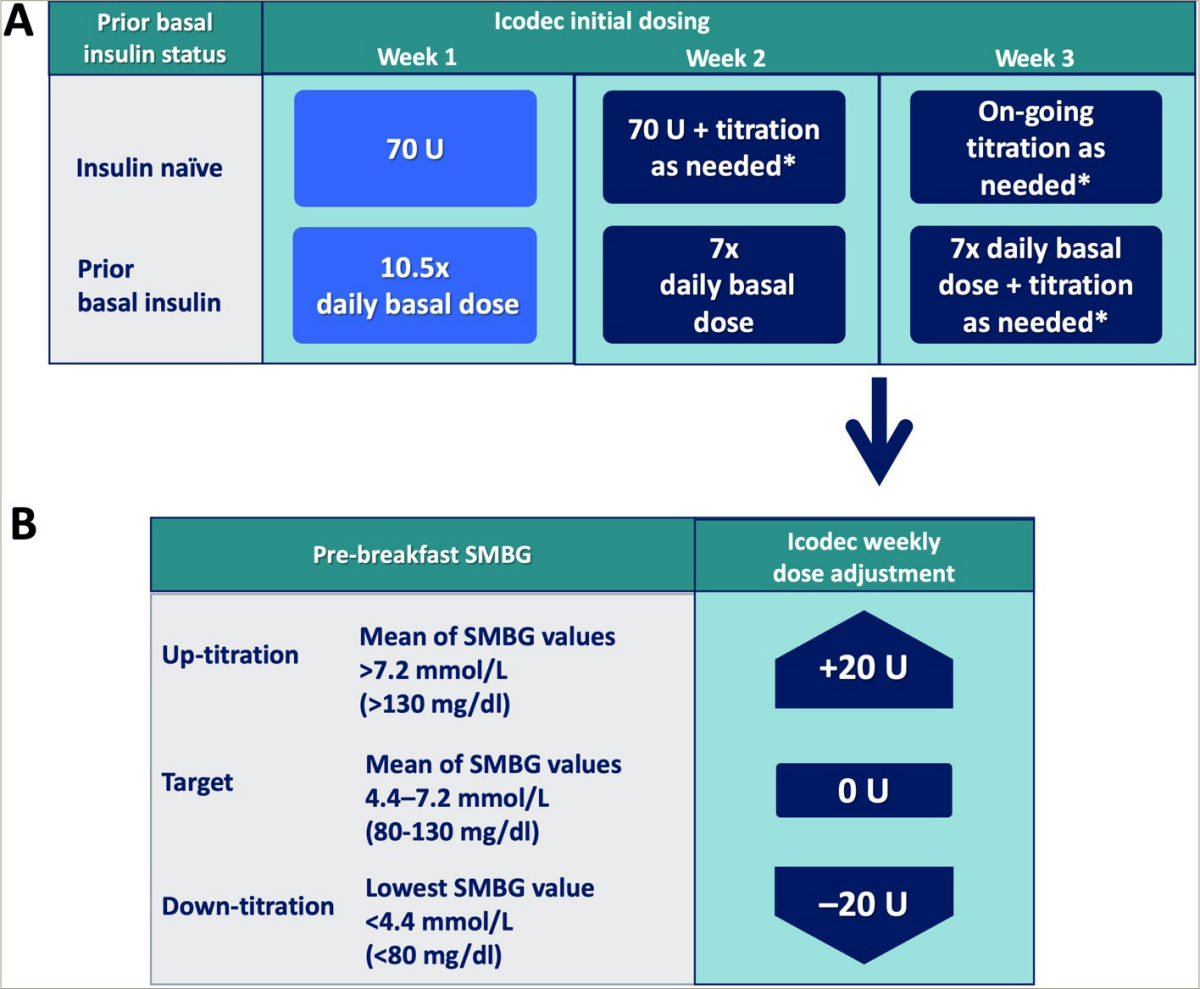
Icodec initial dosing (A) and weekly titration algorithm (B)

*Titration as per *Figure 2B*.

*Weekly dose adjustment is based on three pre-breakfast SMBG values, measured 2 days prior to and on the day of titration. If any of the three pre-breakfast SMBG values are below the lower limit of the target range, titration is based on the lowest recorded value. If all three SMBG values are above the lower limit of the target range, titration is based on the mean of the three measurements.*

*SMBG = self-measured blood glucose.*

*Adapted from Philis-Tsimikas A, et al. 2022.* ^7^

In summary, insulin icodec offers similar or better glycaemic efficacy compared with daily basal insulin in type 2 diabetes, with good tolerability and encouraging safety results related to hypoglycaemia. Although important clinical questions remain, reducing the number of basal insulin injections from 365 to 52 administrations per year may be a significant innovation in insulin management since its discovery more than a 100 years ago.
